# Optimal Hot-Dipped Tinning Process Routine for the Fabrication of Solderable Sn Coatings on Circuit Lead Frames

**DOI:** 10.3390/ma13051191

**Published:** 2020-03-06

**Authors:** Ting Yin, Nan Xiang, Guangxin Wang, Baohong Tian, Wanting Sun, Xiaoyu Zhang

**Affiliations:** 1 School of Materials Science and Engineering, Henan University of Science and Technology, Luoyang 471023, China; yinting0605@hotmail.com (T.Y.); bhtian007@163.com (B.T.); 2 School of Materials Science and Engineering, Harbin Institute of Technology, Harbin 150001, China; sunwt_hit@126.com; 3 School of Environmental and Material Engineering, Yantai University, Yantai 264005, China; zhangxiaoyu@ytu.edu.cn

**Keywords:** hot-dipped tinning process routine, solderable coating, intermetallic compounds, lead frame

## Abstract

Previous studies merely focus on the hot dipping properties of lead frame materials used in electronic industry. Yet, the environmentally friendly and cost-efficient traits of hot-dipped tinning process make it a possible promising surface modification technique compared with electroplating. As a result, the optimal hot-dipped tinning process routine is proposed in this paper. The hot-dipped tinning process of four different types of copper foils (C11000, C19400, C19210, and C70250), pretreatment parameters, mechanical properties of Cu substrates, thickness of IMC (intermetallic compound) layers and coatings, and microstructure of coatings were investigated to determine the copper substrate suitable for hot-dipped tinning and the optimized tinning procedures. The results indicate that a proper increase in alloying elements (e.g., Cu-Fe-P series alloys) towards Cu substrate leads to a decrease in hot dipping performance. The proper process routine is determined as alkaline cleaning→water scrubbing→accelerant solvent dipping→drying→hot-dipped tinning→cooling. The appropriate dipping temperature range is 260 to 280 °C, which assists to maintain acceptable micro hardness (i.e., maintaining at least 95% of the original hardness). The optimal dipping time should be set as 6–10 s. The proposed hot-dipped tinning process routine may present a guideline for the fabrication of tin coating in electronic industry.

## 1. Introduction

Hot-dipped tinning process is a surface modification technique to fabricate metallic coatings by dipping pretreated workpieces into molten metal bath [1]. Coatings may prevent the corrosion of substrates or improve the conductivity and abrasion performance [2]. The substrates in this process are commonly made from steel or copper. The coating materials are typically metallic materials with lower melting points, such as Al, Zn, Pb, Sn, and so forth [3,4].

In electronic industry, Sn is widely used as the protective or solderable coating material for electronic connecters, lead frames in integrated circuit plates, and so forth. Sn can be deposited on the surface of metallic substrates to get dense metallic coatings by employing electroplating, hot dipping, and chemical plating processes. Therefore, electroplating is mostly used due to the uniform and adjustable thickness distribution of coating layers. Yet, the drawbacks of this process are also obvious, such as the complicated technological procedures, high cost, severe environment pollution, and so forth. Comparatively, the hot-dipped tinning process has the advantageous of the simple procedures, sound solderability of coatings, minor pollution, and low cost [5]. Considering the urgent requirements of environmental protection and cost control nowadays, the hot dipping process may become a promising tin plating technique in the future.

Currently, most researchers concentrate on the interfacial reactions during the hot tinning process, such as the phase transformation, the nuclear kinetics, and the evolution in morphology of intermetallic compounds (IMCs). For example, Lee et al. [6] prepared a joint assembly constituted by solder, intermetallic compounds (IMCs), and copper, which experienced a thermal aging test. The morphology at the interface of the composite was observed and the thickness of IMC layer was measured. The characteristics of phase distribution were analyzed. Zhang et al. [7] investigated the growth characteristics and formation mechanisms of the Cu6Sn5 phase, i.e., the intermetallic compound (IMC) phase, at the Sn–Cu interface. A hexagonal-rod-type growth mechanism for Cu6Sn5 phase was proposed on the basis of its anisotropy in surface energy and roughness, which explained the growth mechanism of IMC phase and provided a scientific basis of Cu6Sn5 orientation design. The IMC growth during solid-state aging at 125, 150, and 170 °C for four-solder alloy on Cu substrate was studied by Lee et al. [8], and the morphology, kinetics, and thermodynamics during this process were examined. Furthermore, Zou et al. [9] investigated the morphologies and orientation relationships of Cu6Sn5 grains formed between Sn and Cu single crystals. The evolution pattern of Cu6Sn5 grains and the kinetics of IMCs growth was measured. Buresch et al. [10] studied the effects of oxidation of Sn in hot-dipped tinning process on contact resistance and solderability of Cu substrate. The differences between hot-dipped tinning and electrolytic tinning were compared. Gagliano et al. [11] dipped copper specimens into molten tin for 1 and 2 s at 240–300 °C to determine the nucleation kinetics of η phase (Cu_6_Sn_5_). The typical inverse C type behavior was observed with a maximum nucleation rate occurring at an intermediate temperature. Ohki et al. [12] investigated the growth of IMC layer in hot-dipped tinning process and its adhesive strength. The growth regulation of IMC layer was presented. Takaku et al. [13] focused on the morphologies and growth of Cu_3_Sn and Cu_6_Sn_5_ intermetallic compounds between molten Sn solder and Cu substrate. It is found that the thickness of IMC layer decreases with the Sn content decreasing and the growth of these phases basically obeys the parabolic law. Li et al. [14] explored the interfacial reaction kinetics between molten Sn-58Bi solder and Cu substrates. The elemental additions of 1–2 wt.% Al, Cr, Cu, Si, Zn, Ag, Au, Pt, and Nb into the basic Sn-58Bi solder were attempted to produce a barrier layer to slow down IMC growth.

Based on aforementioned studies, the adhesive strength between Sn coating and Cu substrate, the mechanical properties, and electrical properties of Sn coatings were investigated to benefit the industrial application of hot-dipped tinning process. For instance, Mendala [15] investigated the failure possibility of partly coating in Sn or Zn baths subjected to external stress. It was indicated that the chemical composition and temperature of metallizing bath affect the mechanical performance of parts. Xiang et al. [16] studied the wetting force between Cu substrates and Sn coatings, and preliminarily evaluated the dipping properties. The wetting ability of Sn coatings was considered to be related with pretreatment conditions. Braunovic [17] focused on the effects of IMC phases on electrical and mechanical (microhardness) properties of tin-plated copper conductors and connectors. It showed that IMC phases adversely affects these properties of tin-plated conductors and connectors, which was demonstrated in the case of flexible tin-plated copper connectors used in different types of electrical applications. Chang et al. [18] studied the adhesion strength of Sn-9Zn-0.5Ag solder hot-dipped on Cu substrate. The influences of Ag content, dipping time, and dipping temperature were discussed. Similarly, Lee et al. [19] investigated the influence of IMC growth on the adhesive strength of solder joints. The types of solders were found to be related to the temperature and the adhesive strength of solder joints. Zhang et al. [20] measured the fracture behaviors of the interfacial Cu–Sn IMC layers induced by deformation of the Cu substrates, the fracture morphologies were observed and the yield behavior of Cu substrates was considered to be the major influencing factor on the fracture of IMC layers. Lee et al. [21] determined the adhesive strength, shear strength, and fractographic morphology of Sn–Ag-coated Cu wires. They pointed out that the adhesive strength and the shear strength of solder joints decreases significantly with short-term thermal storage. Moreover, some researcher discussed the fretting corrosion, electronic performance, friction, and abrasive performance of hot-dipped Sn contactor. For instance, Park et al. [22] analyzed the effects of displacement amplitude, normal load, frequency, relative humidity, and temperature on fretting corrosion behavior of hot dipped tin coating and examined the fretted surface.

Researches on the comprehensive evaluation for the effects of technological parameters on coating quality and the consequent optimal process routine is still limited, which restricts the further application of this process. Microelectronics industry now develops toward the orientation of high performance and high integration. The volume of electron devices and the dimensions of solder joints become smaller and smaller. Then the requirements for mechanical, electronical, thermal performance, and the reliability of Sn coatings are more stringent.

As to the performance of coating materials, Sn-Pb alloys have widely been used in the field of electronic packaging for many years [23]. The addition of Pb can prohibit the formation of whiskers effectively. However, the use of Pb in electronics was prohibited as Sn-Pb alloys cause serious environment pollution [8]. Therefore, the interfacial reactions between lead-free solders (Sn-Ag, Sn-Ag-Cu, and Sn-Cu) and substrates as well as their mechanical properties have attracted much attention [24,25]. Actually, Sn-Pb solders also exhibit remarkable performance deficiencies, such as inferior creeping property and lower shear strength, As a result, Sn-Pb alloys cannot meet the requirements of high integration and reliability and thus cannot be adopted as coating material in the hot-dipped tinning process.

On the other hand, to fabricate hot-dipped coating layer of Cu foils used for electron devices, the uniformity of coating layer, the strength of substrate after hot dipping, the thickness of coating layer, and the conductive whisker should be controlled properly. It involves overall consideration of the selection of substrates, the optimization of pretreatment parameters, and hot dipping parameters. The choice of substrates depends on the types, microstructure, adhesive strength of IMCs, and the amount of whiskers. The pretreatment parameters will influence the surface quality and the wettability of substrates. The hot dipping parameters are related to the thickness of coatings and IMC layers, the mechanical properties of substrates, and the adhesive strength. Therefore, it is essential to develop a sophisticated hot-dipped tinning process routine, which can adapt with industrial lead frame materials.

The purpose of this paper is to develop an optimal hot-dipped tinning process routine and evaluate its applicability on different lead-free lead frame materials. Concretely, effects of pretreatment schemes on surface morphology of hot-dipped Cu foils and the element distribution at coating interface are investigated at first. Then, effects of hot dipping parameters on the microhardness of Cu substrates are discussed. The thickness variation of coating layer and IMC layer under different dipping parameters for different types of Cu foils are determined. Microstructure at the interface between Cu substrate and Sn coating layer is examined. Thereafter, appropriate hot-dipped tinning process routine is developed.

## 2. Hot-Dipped Tinning Experiments for Copper Foils

### 2.1. Introduction to Hot-Dipped Tinning Process

**Figure 1:**
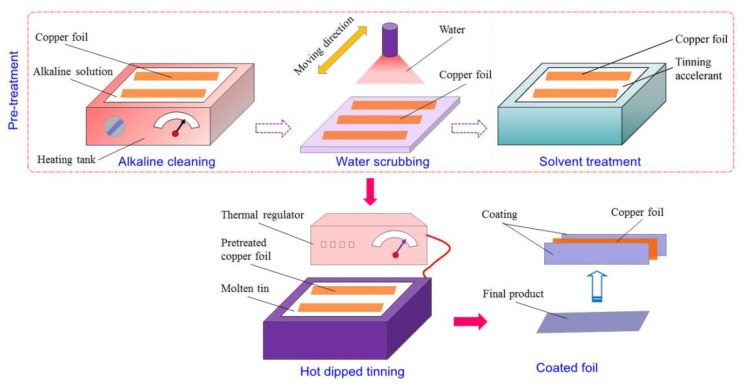
Process procedures of hot-dipped tinning.

Detailed process procedures of hot-dipped tinning process for copper foils are illustrated in Figure 1. Pretreated copper foils were dipped into molten tin bath for appropriate time; then, the phase transformation of L→η→ε occurs at the interface between molten Sn and Cu substrate [26,27], and the chemical composition of η phase and ε phase is Cu_6_Sn_5_ and Cu_3_Sn, respectively; consequently, the tin layer firmly adheres to copper substrate like electroplated coatings or chemical plated coating. Solvent treatment is implemented to improve the wettability of copper substrates, which may enhance the contact between molten tin and copper substrates and promote the formation of IMCs. Due to the simple process procedures, short lead time, and environmentally friendly characteristic, the hot-dipped tinning process increasingly becomes a potential coating method to take place of electroplating and chemical plating processes.

Technological parameters of hot-dipped tinning process mainly cover components of accelerant and molten tin, dipping temperature, dipping time and so forth. To get the optimal process routine of hot-dipped tinning process, the combined control of aforementioned factors is essential. The thickness of coatings, thickness of IMC layer, and the microstructure of coatings are commonly used to evaluate the hot tinning capability.

### 2.2. Materials and Characterization

The substrate materials used in the experiments are four types of cold-rolled copper foils, the trademarks of which are C70250, C19400, C19210, and C11000. All these copper foils are manufactured by Chinalco Luoyang Copper Group Co. Ltd. (Luoyang, China). The dimensions and mechanical properties of copper foils are shown in Table 1. The chemical compositions of copper alloys are presented in Table 2. It can be seen that the micro-addition of alloying elements leads to dramatic increase of mechanical properties of Cu alloy.

The tin blocks used in experiments were produced by Yunnan Tin Co. Ltd. with grade mark Sn99.90AA. The content of Sn is 99.970%, which is in accordance with Chinese standard GB/T728- 2010. In experiment, tin blocks were cut, put into stainless crucible, and heated to a molten state. The pretreatment of four types copper foils (C70250, C19400, C19210, and C11000) includes alkaline cleaning, water scrubbing, and solvent treatment. All of the chemical reagents used in this paper are AR analytical pure. NaOH solution and distilled water were used in alkaline cleaning and water scrubbing, respectively. The molar concentration of accelerant solvent is listed in Table 3. Hot-dipped tinning process was carried out self-made devices which consist of stainless crucible, resistance heater, and temperature controller. The temperature of molten tin was measured by thermo couple, and the temperature control accuracy is ±2 °C.

Hot-dipped four types copper foils Cu foils were ground and polished by abrasive paper (grade mark: 400–2000). DNW1.5 water-soluble diamond paste and distilled water were added continuously during polishing to prevent the occurrence of scratch. The as-polished specimens were rinsed by absolute ethyl alcohol and corroded by mixed solution composed of 4% HNO_3_ and 2% HCl.

The microstructure of specimens was observed by JSM-5610LV scanning electron microscope (SEM) equipped with energy dispersive spectrum (EDS) (Japan Electronics Corporation (JEOL), Akishima, Japan). Three to nine sample regions were selected for each dipping temperature and dipping time. The pixel transfer method was employed to determine the thickness of IMC layer (*δ*_IMC_) and coating layer (*δ*) [28].

The micro-Vickers hardness of hot-dipped specimens was measured by HVS-1000A microhardness tester (Laizhou Huayin Testing Instrument Co., Ltd., Laizhou, China). The applied force is 50 gf with duration of 5 s. 3–5 measurement points were selected for each specimen to calculate the average microhardness.

### 2.3. Schemes of Pretreatment Experiments

Before hot-dipped tinning process, pretreatment experiments were conducted to explore appropriate procedures and parameters suitable for subsequent hot-dipped tinning process. The pretreatment parameters are shown in Table 4. Sample 1 was taken as a reference sample without any pretreatment. Sample 2 and sample 3 were 10 wt% HCl acid pickled for 4 min at followed by alkaline cleaning to remove the oxidation layer. This duration was selected as 4 min in this experiment. The pretreated specimens were hot-dipped for 6 s at 280 °C to observe the effects of different pretreatment parameters.

### 2.4. Schemes of Hot-Dipped Tinning Experiments

Cu foils pretreated by employing optimal pretreatment parameters were hot-dipped in accordance to the schemes presented in Table 5. Crossover experiments were conducted at each dipping temperature for each dipping time.

## 3. Results and Discussion

### 3.1. Optimization of Pretreatment Parameters

**Figure 2:**
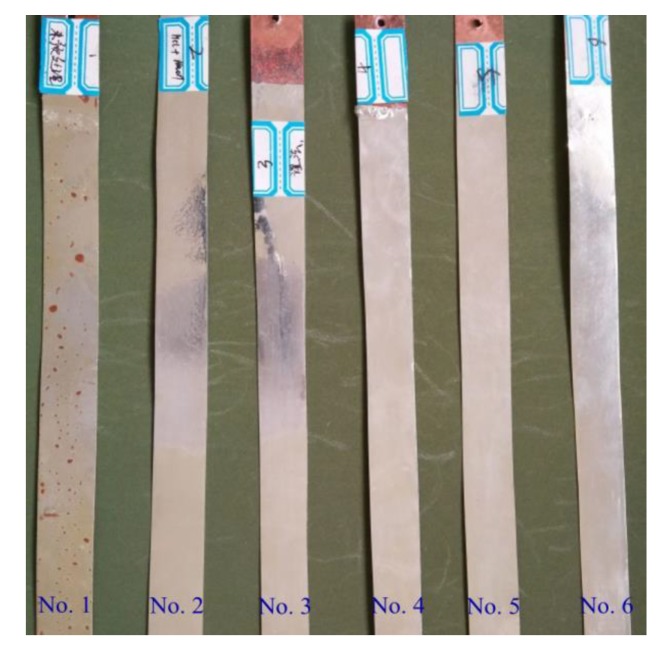
Hot-dipped specimens processed by using different pretreatment parameters.

Pretreatment experiments of C11000 foils were conducted to determine pretreatment parameters that are suited to hot-dipped tinning processes. After hot dipping, the six samples were exposed in air for 120 h to observe the surface morphology. Figure 2 provides hot-dipped specimens pretreated by different approaches. The six specimens shown in Figure 2 from left to right were treated by parameter No. 1 to No. 6 listed in Table 4, respectively. Hot tinning parameters for all these specimens kept constant, i.e., dipping temperature of 280 °C and dipping time of 6 s. In the view of surface appearance, plenty of pitting appears on the surface of sample 1 (without any pretreatment), indicating the necessity of pretreatment before hot dipping. Samples 2 and 3 were treated by acid pickling and show apparent nonuniform distribution of Sn coatings. As a result, acid pickling before hot dipping was useless. Samples 4–6 were alkaline cleaned, solvent treated, and dried under different conditions, i.e., quick dried (dried with a dryer at 150 °C for 10 min), natural dried (dried in air naturally), and undried (hot dipping without drying after pretreatment). The quick dried specimen (i.e., sample 4) shows inferior degree of finish compared with natural dried specimen (i.e., sample 5) and undried specimen (i.e., sample 6). Because ammonium salt and muriatic acid will volatilize during drying procedure the inorganic salt may adhere to Cu foils and will not melt at elevated temperature, which may result in the unstable adhesion of surface coatings. Besides, accelerant solvent may react with tin oxidations to reduce the generation of tin oxidations. Then few tin oxidations will adhere to Cu foils and the surface roughness can be improved.

**Figure 3:**
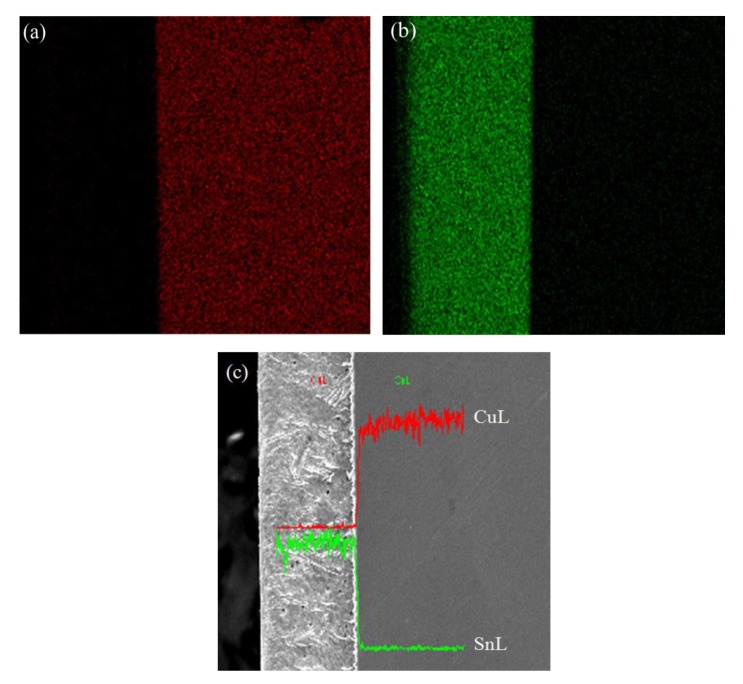
Element distribution of hot-dipped specimen processed with pretreatment parameter No.6: (**a**) distribution of Cu, (**b**) distribution of Sn, and (**c**) energy-dispersive spectroscopy (EDS) spectra and elements quantification.

Sample 6 with the best surface quality was selected to conduct the EDS analysis along its section direction. Figure 3 shows the element distribution of coating and substrate. Cu atoms concentrate in the right side of the Figure 3a, which represents the substrate, whereas Sn atoms concentrate in the left side of the Figure 3b, which refers to the coating. It is clear that the coating thickness is uniform, which demonstrates the feasibility of pretreatment parameters used for sample 6. Also, it can be considered to be applicable for the technological procedures covering alkaline cleaning, water scrubbing, accelerant solvent dipping, and hot-dipped tinning. Figure 3c is the EDS spectra and elements quantification of sample 6. It can be seen from the energy spectrum analysis chart that the green SnL polyline represents the element content distribution of the Sn element in the horizontal scale direction. The red CuL polyline represents the element content distribution of the Cu element in the horizontal ruler direction. The red CuL polyline in the silver part is lower and the green SnL polyline is higher, indicating that Sn is the main element in this part, and the Cu content is almost negligible. The curve distribution of the gray part is exactly opposite to that of the silver part, indicating that the main element in this area is Cu, and the content of Sn can be ignored. Both of the bright white parts of the polyline are at the highest peak, indicating that the content of both elements in this area is very high, so this area is the IMC area.

### 3.2. Effects of Hot Dipping Parameters on Microhardness of Cu Substrate

**Figure 4:**
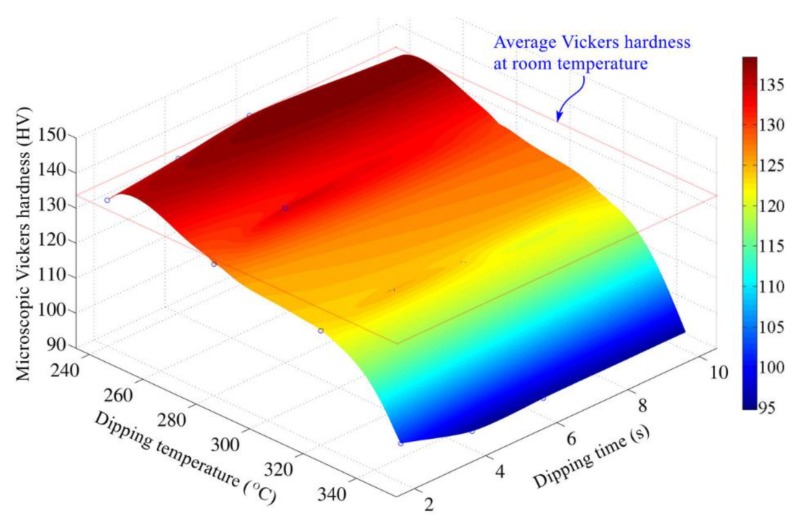
Variation of Vickers hardness of Cu substrates with dipping temperature and dipping time for C11000 foils.

To study the effects of hot tinning parameters (i.e., dipping temperature and dipping time) on mechanical properties of Cu substrate, the micro-Vickers hardness of C11000 foils processed under different hot tinning parameters were measured. Figure 4 presents the variation of average micro-Vickers hardness with dipping parameters. The initial micro-Vickers hardness of C11000 foils is determined as 133.4 HV, which is taken as the reference to compare with hot-dipped foils. It indicates that micro-Vickers hardness of Cu foils does not vary with dipping time within the range of 2–10 s. However, with the increase of dipping temperature, the micro-Vickers hardness of Cu foils show different variation tendency. In the range of 240 to 280 °C, the maximum decrease of microhardness of Cu foils is 4.3%, which is negligible. When dipping temperature exceeds 280 °C, the microhardness of Cu foils starts to reduce sharply. For instance, in the case that dipping temperature increases to 320 °C, the microhardness of Cu foils reduces by 6.4%–10.0%. In the case that dipping temperature increases to 350 °C, the microhardness of Cu foils reduces by 24.2%–28.6%.

The recrystallization of Cu substrates may be responsible for the decrease of microhardness. It is well known that the recrystallization temperature of metal (*T*_rec_) and its melting point (*T*_mel_) commonly have the following relationship, *T*_rec_ (K) = 0.3–0.4*T*_mel_ (K) [18]. The melting temperature of Cu is 1083 °C; then, its recrystallization temperature probably stays in the range of 200 to 280 °C. In the condition that dipping temperature exceeds the recrystallization temperature, distortionless crystal nucleus appears and then grows up on the metallic substrate. As a result, the grains, which deformed during rolling process, are transformed to new equiaxed grains. The microstructure and mechanical properties of Cu substrate change remarkably. The strain hardening is eliminated. When the dipping temperature is higher than 320 °C, then recrystallization of Cu occurs, which is actually the growth of distortionless crystal nucleus in the deformed Cu substrate. The recrystallization eliminates strain hardening generated in the cold rolling process of Cu foils and consequently lowers down its microhardness.

### 3.3. Variation of Coating Thickness and IMC Layer Thickness with Hot Dipping Parameters

According to the Cu–Sn binary phase diagram [26,27], phase transformation of L→η→ε occurs at the interface between molten tin and Cu substrate within the temperature range of 240 to 350 °C. The experimental results obtained by Li et al. [14] demonstrated that η phase mainly appears at the interface in Cu-Sn system and the chemical composition of η phase is Cu_6_Sn_5_. Therefore, the Cu-Sn IMC layer is mainly consisted of Cu_6_Sn_5_.

**Figure 5:**
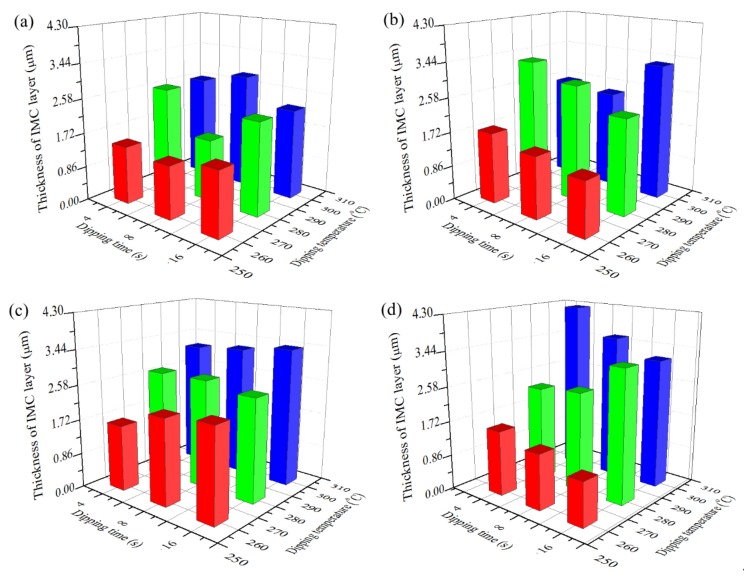
Variation of IMC layer thickness with dipping temperature and dipping time for different types of copper foils: (**a**) C70250 foils, (**b**) C19400 foils, (**c**) C19210 foils, and (**d**) C11000 foils.

Figure 5 shows the variation of IMC layer thickness with dipping temperature and dipping time after the hot dipping of Cu foils. With the dipping temperature increasing, thickness of IMC layer formed on the surface of four types of Cu foils all increases gradually. Take specimens dipped for 4 s as an example, when dipping temperature increases from 260 °C to 300 °C, IMC layer thickness of C70250 foils increases from 1.4 μm to 2.6 μm, IMC layer thickness of C19400 foils increases from 1.7 μm to 2.5 μm, IMC layer thickness of C19210 foils increases from 1.6 μm to 3.1 μm, and IMC layer thickness of C11000 foils increases from 1.6 μm to 4.3 μm. This occurs because as the mechanical properties of Cu substrates are decreasing, the IMC layer thickness gradually increases; whereas, with concentration of alloying elements in Cu substrates increasing, the mechanical properties of Cu substrates increase. Consequently, IMC layer thickness decreases with the increase of alloying elements addition. The effects of dipping time on IMC layer thickness are much less than dipping temperature.

**Figure 6:**
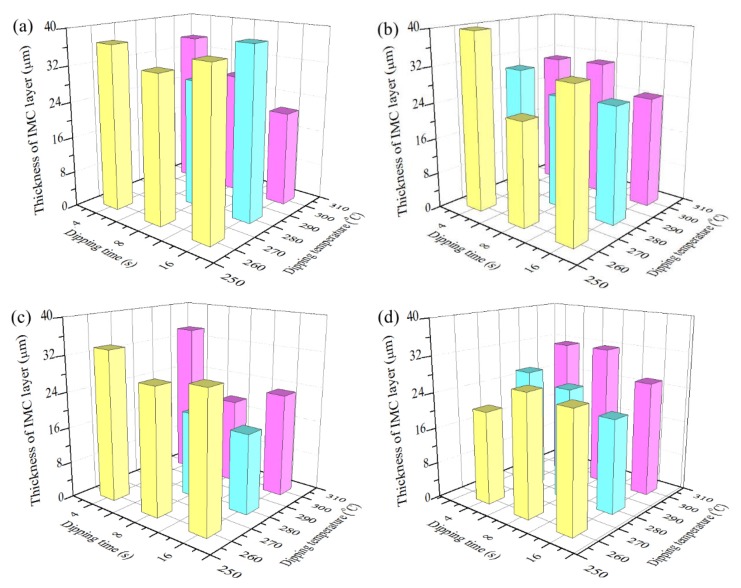
Variation of coating thickness with dipping temperature and dipping time for different types of copper foils: (**a**) C70250 foils, (**b**) C19400 foils, (**c**) C19210 foils, and (**d**) C11000 foils.

Figure 6 shows the variation of coating layer thickness with dipping temperature and dipping time after hot dipping. The coating layer thickness of the C70250, C19400, and C19210 foils decreases with the increase of dipping temperature. Yet, the coating layer thickness of C11000 foils increases with the increase of dipping temperature. Effects of dipping time on layer thickness of four types of coatings are irregular. However, the dipping time less than 6 s will be impracticable and result in the splatter of molten tin; dipping for too long time lead to the recovery and recrystallization of Cu substrates, which softens Cu foils and lowers down theirs performance in use.

Through the overall consideration of coating layer thickness, IMC layer thickness and feasibility of practical production, dipping temperature of 260–300 °C, and dipping time of 6–10 s are reasonable. In this condition, coating defects such as oxidation of molten tin, insufficient flowability of liquid tin, inferior mechanical properties of Cu substrates can be properly avoided.

### 3.4. Coating Layer Structure of Different Cu Foils

**Figure 7:**
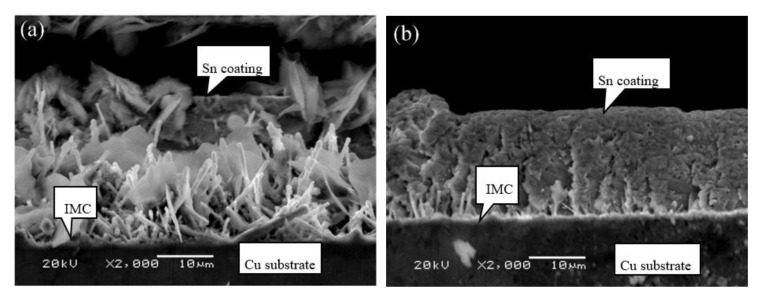

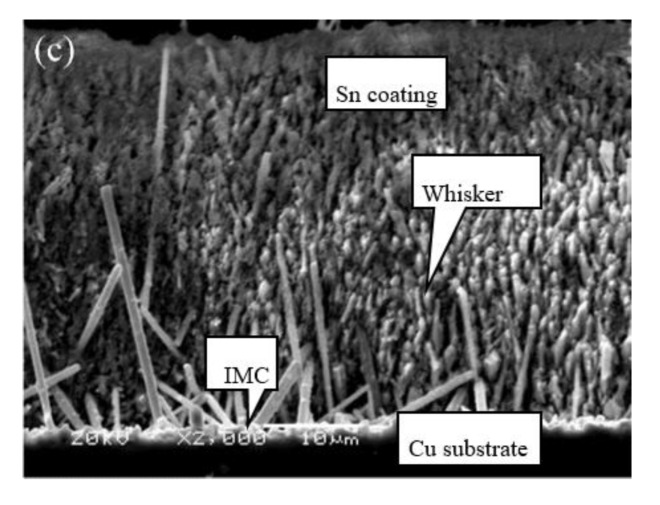
Cross-sectional microstructure of C70250 foils coated at 280 °C with different dipping time: (**a**) 4 s, (**b**) 8 s, and (**c**) 16 s.

Cross-sectional microstructures of C70250 foils coated for different dipping time at 280 °C are presented in Figure 7. Apparently, the surface of coating is rough and uneven, and the morphology of IMC layer varies significantly with the extension of dipping time. When dipping time increases to 16 s, tin whisker grows throughout the whole coating layer, and the coating thickness became larger. Therefore, the hot dipping performance of C70250 foils is inferior to the other ones. However, elucidation of the mechanism requires further investigation.

**Figure 8:**
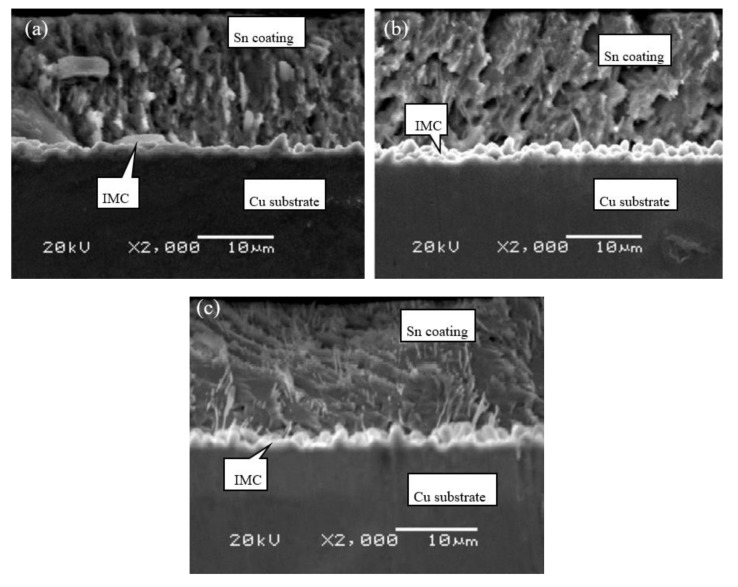
Cross-sectional microstructure of C19400 foils coated at 280 °C with different dipping time: (**a**) 4 s, (**b**) 8 s, and (**c**) 16 s.

The cross-sectional microstructures of C19400 foils coated under different dipping time at 280 °C are shown in Figure 8. Typical columnar crystal characteristic can be observed. Apparently, the hot dipping performance of this alloy is superior to C70250 alloy since the quantity of whiskers dramatically decreases. The whiskers appear only under the condition that dipping time exceeds 16 s. The thickness of coating layer remains almost constant.

**Figure 9:**
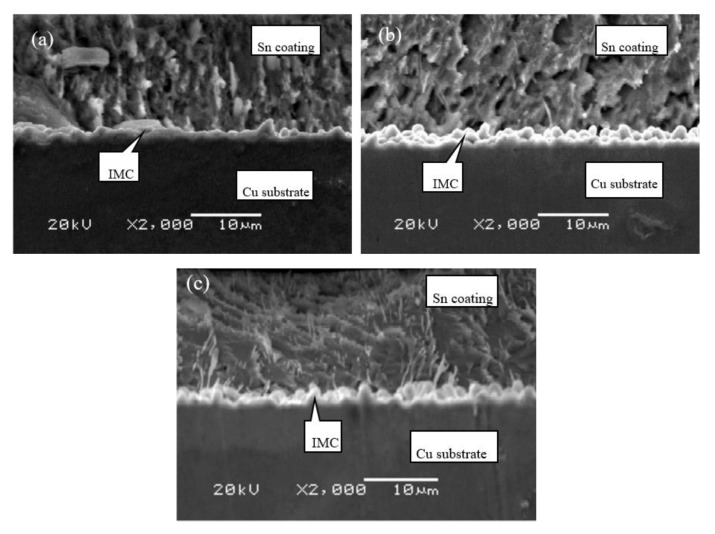
Cross-sectional microstructure of C19210 foils coated at 280 °C with different dipping time: (**a**) 4 s, (**b**) 8 s, and (**c**) 16 s.

The cross-sectional microstructures of C19210 foils coated for different dipping time at 280 °C are shown in Figure 9. The columnar crystal characteristic of C19210 foils, which is similar to C19400 foils, is also observed. The whiskers also appear when dipping time exceeds 16 s. The thickness of coating layer does not change remarkably. Thus, the hot dipping performance of C19210 foils is also preferable as compared to that of C70250 foils.

**Figure 10:**
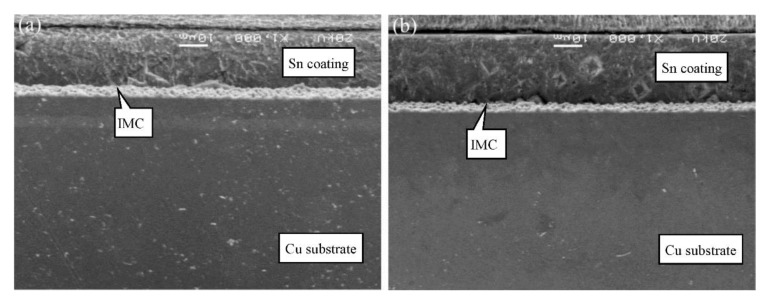
Cross-sectional microstructure of C11000 foils coated at 280 °C with different dipping time: (**a**) 2 s and (**b**) 4 s.

The cross-sectional microstructures of C11000 foils coated for different dipping time at 280 °C are presented in Figure 10. It is apparent that the hot dipping performance of C11000 alloy is superior to the C19400 alloy and C19210 alloy. The thickness of coating layer is uniform. The adhesive interface between Cu substrate and coated tin layer is smooth, indicating a high adhesive strength.

Generally, C11000 foils, containing the least content of alloying elements among all four types of Cu foils, show the best hot dipping performance. The coating layer is uniform and no whisker appears at the interface. The hot dipping performance of C19400 foils and C19210 foils, which are classified as Cu-Fe-P series alloys, are moderate. The thickness of IMC layers is thinner and the corresponding coating layer is uniform. The hot dipping performance of C70250 foils, which is classified as Cu-Ni-Si series alloys, is inferior. It is necessary to modify their alloying element content or adjust the content of molten tin to improve its hot dipping performance.

### 3.5. Optimal Hot-Dipped Tinning Process Routine

**Figure 11:**
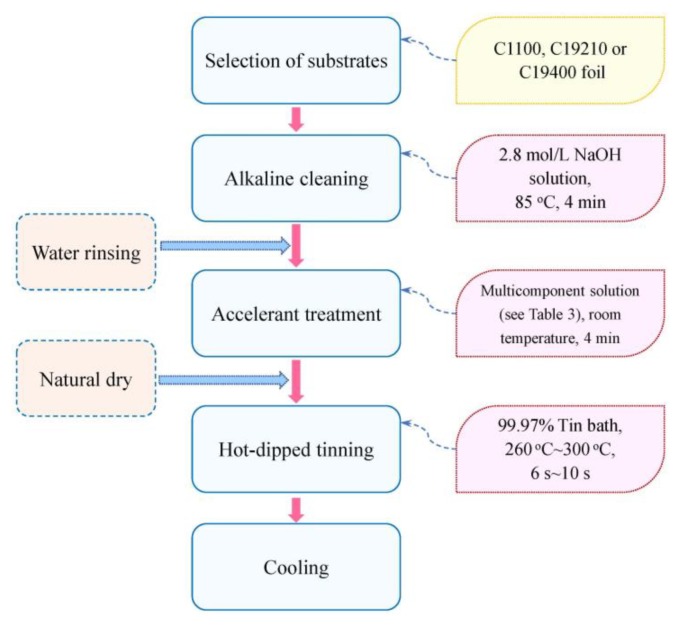
Optimal hot-dipped tinning process routine for copper lead frames used in integrated circuit plates.

The effects of content of Cu substrates, pretreatment parameters, and hot dipping parameters on hot dipping performance should all be taken into consideration when designing the process routine. Based on aforementioned analysis, the optimal hot-dipped tinning process routine for the manufacture of coated Cu foils used as lead frames is determined as follows; alkaline cleaning→water scrubbing→accelerant solvent treatment→drying→hot-dipped tinning→cooling (see Figure 11). Alkaline liquor used in alkaline cleaning procedure is NaOH solution with the concentration of 2.8 mol/L. Alkaline liquor is heated in water bath at 85 °C. The alkaline cleaning procedure lasts for 4 min. Next, the Cu foils are rinsed with distilled water immediately to prevent the deposition of inorganic salts or the grease demulsifying. Subsequently, accelerant solvent treatment is performed to eliminate the oxidation film formed after water scrubbing, and promote the wetting of molten tin to Cu substrate. The chemical composition of mixed solution is presented in Table 3. Solvent treatment is conducted at room temperature and lasts for 4 min. Cu foils after solvent treatment are natural dried. For hot-dipped tinning experiments, tin bath with the concentration of 99.97% is employed. Dipping temperature is set as 260–300 °C and dipping time is set as 6–10 s. C11000 foils, C19400 foils, and C19210 foils are recommended to manufacture coated lead frames through hot-dipped tinning process.

## 4. Conclusions

The pretreatment parameters, mechanical properties of Cu substrates, thickness of IMC layers and coating layers, and microstructure of coatings are investigated in this paper. The Cu substrates suitable to hot-dipped tinning process and the optimal hot-dipped tinning process routine are elucidated. It could be concluded as follows.

(1) The increase of alloying elements in Cu substrates leads to decreasing hot dipping performance. C11000 alloy shows the best hot dipping performance among four types of Cu substrates, i.e., no whisker appears and coating layer is uniform. The hot dipping performance of Cu-Fe-P series alloy, i.e., C19400 alloy and C19210 alloy, is moderate. However, dipping over 16 s may lead to the appearance of whiskers. However hot dipping performance of Cu-Ni-Si series alloy, i.e., C70250 alloy, is inferior. The surface of coating layer is uneven and plenty of whiskers appear.

(2) Appropriate dipping temperature is essential to keep acceptable microhardness. In the case that dipping temperature exceeds 280 °C, recrystallization of Cu substrates occurs, which eliminates the strain hardening of cold-rolled Cu foils and reduces their microhardness. In the case that dipping temperature is less than 280 °C, no recrystallization of Cu substrates occurs, but the flowability of molten tin decreases distinctly. Therefore, the appropriate dipping temperature range is 260 to 280 °C.

(3) The effects of dipping time on coating thickness of Cu foils and IMC layer thickness are irregular. However, too short dipping time is infeasible in practical production; too long dipping time leads to the recovery and recrystallization of Cu substrates and soften substrates. As a result, dipping time of 6–10 s is reasonable.

(4) The optimal hot-dipped tinning process routine is determined as alkaline cleaning→water scrubbing→accelerant solvent treatment→drying→hot-dipped tinning→cooling. Using this process routine, hot tinned Cu foils without macroscopic defects, with uniform coating morphology, and preferable mechanical properties can be obtained.

## Figures and Tables

**Table 1:** Dimensions and mechanical properties of copper foils used in experiments.

Category	Grade of Copper Foil			
	C70250	C19400	C19210	C11000
Heat treatment state	TM02	H00	H02	TM02
Thickness, *δ*_0_ (mm)	0.640	0.254	0.200	0.200
Size (mm × mm)	10 × 44	10 × 300	10 × 82	10 × 200
Tensile strength, *σ_b_* (MPa)	658	431	379	350
Yield strength, *σ_s_* (MPa)	/	400–402	/	245
Elongation, *ε* (%)	15.0–16.0	7.5	19.0	8.0
Hardness, *H* (HV)	204	138	121	133
Surface roughness, *R_a_* (μm)	0.082	0.069	0.075	≤ 0.300

**Table 2:** Chemical compositions of copper foils used in experiments.

Materials	Cu (%)	Fe (%)	P (%)	Zn (%)	Sn (%)	Ni (%)	Si (%)	Mn (%)	Mg (%)
C70250	96.67	/	/	/	/	2.0	0.6	<0.1	0.17
C19400	97.54	2.3	0.028	0.1	/	/	/	/	/
C19210	99.8	0.11	0.032	/	/	/	/	/	/
C11000	99.97	0.005	/	0.005	0.005	/	/	/	/

**Table 3:** Molar concentration of each component in accelerant flux.

ZnCl_2_ (mol/L)	NH_4_Cl (mol/L)	NaCl (mol/L)	HCl (mol/L)
1.80	0.58	1.14	0.08–0.17

**Table 4:** Parameters used in pilot experiments before the hot dipping experiments.

No.	Duration of each Procedure (min)			Dry Method
	Acid Pickling	Alkaline Cleaning	Dipping in Accelerant	
1	/	/	/	/
2	5	4	4	Natural dry
3	5	4	4	/
4	/	4	4	Quick dry
5	/	4	4	Natural dry
6	/	4	4	/

**Table 5:** Experimental parameters for hot-dipped tinning.

Materials	Dipping Temperature, *T* (°C)	Dipping Time, *t* (s)
C70250	260, 280, 300	4, 8, 16
C19400	260, 280, 300	4, 8, 16
C19210	260, 280, 300	4, 8, 16
C11000	240, 260, 280, 300, 350	2, 4, 8, 16
